# Retraction: Extracellular vesicle-derived microRNA-410 from mesenchymal stem cells protects against neonatal hypoxia-ischemia brain damage through an HDAC1-dependent EGR2/Bcl2 axis

**DOI:** 10.3389/fcell.2026.1868597

**Published:** 2026-05-22

**Authors:** Frontiers Editorial Office

The journal retracts the 11 January 2021 article cited above.

Following publication, concerns were raised regarding the integrity of the images in the published figures. The authors failed to provide a satisfactory explanation during the investigation, which was conducted in accordance with Frontiers’ policies.

This retraction was approved by the Chief Editors of Frontiers in Cell and Developmental Biology and the Chief Executive Editor of Frontiers. The authors have not responded to correspondence regarding this retraction.

